# Supplementary material for: Native Ambient Mass Spectrometry of an Intact Membrane Protein Assembly and Soluble Protein Assemblies Directly from Lens Tissue
Source: Angew Chem Weinheim Bergstr Ger. 2022 Jun 21;134(31):e202201458. doi: 10.1002/ange.202201458 (PMC10946450; doi:10.1002/ange.202201458)
Supplement: Supplementary file 1 — Supporting Information [file ANGE-134-0-s001.pdf]

Angewandte Chemie
Eine Zeitschrift der Gesellschaft Deutscher Chemiker

Supporting Information

Native Ambient Mass Spectrometry of an Intact Membrane Protein Assembly and Soluble Protein Assemblies Directly from Lens Tissue

*O. J. Hale, H. J. Cooper**

SUPPORTING INFORMATION

Experimental

Materials

MS-grade water was purchased from Fisher Scientific (Loughborough, UK). HPLC-grade ammonium acetate was bought from J.T. Baker (Deventer, Netherlands). The detergent C_8E_4 was bought from Sigma-Aldrich (Gillingham, UK). Mass spectrometer calibration was performed with FlexMix (Thermo Fisher, San Jose, CA)

Solvent systems consisted of 200 mM aqueous ammonium acetate with C8E4 detergent added as required to give concentration at approx. 2x CMC. No organic solvents were used. Nitrogen (>99.995%) and helium (>99.996%) gases used on the mass spectrometer were obtained from BOC (Guildford, UK).

Eye lens tissue

Whole, fresh sheep eyes were bought from DissectUK (Birmingham, UK). Eyes were harvested and transported with cold packs for dissection the same day. On receipt, eyes were dissected, and the lenses were extracted. Lenses were placed on aluminium foil and snap frozen in liquid nitrogen. All tissue was stored at -80 °C, sectioned at -22 – 24 °C to a thickness of 20 μm with a CM1810 Cryostat (Leica Microsystems, Wetzlar, Germany) and thaw mounted to glass microscope slides. The fresh frozen tissue adheres poorly to glass, particularly that of the lens nucleus. A previously published methanol mounting technique could not be used due to the risk of alcohol denaturing and redistribution of proteins. Sections were stored at -80 °C, and thawed at room temperature 30 mins before use. Sections were not washed prior to analysis to avoid protein delocalisation and potential structural disruption.

Nano-DESI MS

A home-built nano-DESI ion source was attached to an Orbitrap Eclipse mass spectrometer (Thermo) equipped with the HMR^n option. Briefly, an XYZ-stage (Zaber Technologies Inc., Vancouver, Canada) was mounted at the mass spectrometer inlet. Primary and sampling capillaries were flame-pulled from fused silica tubing (O.D. 275 μm, I.D. 75 μm prior to modification) and cut to a final outer diameter of approx. 100 μm. The sampling capillary was positioned approx. 0.5 mm within the mass spectrometer inlet to aspirate solvent with the inlet vacuum. The exit of the sampling capillary was not flame-pulled but had the coating removed. Solvent was delivered through the solvent capillary by a 10 mL gas-tight syringe (Hamilton, Reno, NV) and a liquid junction was formed between the two capillaries. High voltage was provided by a wire from the mass spectrometer power supply connected directly to the syringe needle and optimized for ion intensity and spray stability (typically between 0.8 and 1.5 kV).

Stage movement was controlled directly by Zaber Control (Zaber Technologies Inc.) or automated by custom software written in LabVIEW (NI, Austin, Texas). *In situ* intact protein profiling and top-down mass spectrometry analyses were performed by positioning thin tissue sections underneath the nano-DESI probe and scanning the probe across the surface at between 1 and 50 μm/s, depending on application. A relay connected to an Arduino Uno v3 microcontroller triggered contact closure to signal the start of a new line scan in MSI experiments.

Mass spectrometry imaging

Two individual lenses were imaged in duplicate. For each analysis, the solvent flow rate was set to 1.9-2 μL/min and the electrospray voltage was 1.0-1.3 kV. The nano-DESI probe was moved laterally at 50

μm/s with line spacing of 200 μm. The delicate nature of the lens tissue and its weak adherence to the glass surface necessitated rapid probe movement to limit removal of bulk tissue during analysis. The mass spectrometer inlet was set to 275 °C. SDV was set to 180 V and SCV was set to 17%. The ion routing multipole (IRM) chamber pressure was set to 20 mTorr. m/z analysis was performed in the orbitrap analyser at a resolution of 7500 at m/z 200 (transient length of 16 ms). The duration for each imaging experiment was approximately four hours.

Ion images were produced by conversion of Thermo raw files for each line scan to a single imzML file by Firefly (v.3.2.0.23, Prosolia, Inc., Indianapolis, IN). Pixels in the ion images were 200 x 200 μm (representing 0.04 mm^2 of tissue), composed of the sum of 4 s of data each. Ion images were processed in MSiReader[1] and had 1x linear interpolation applied, with TIC normalisation and a linear intensity scale. Protein ions form in multiple charge states when analysed by nano-DESI.

Top-down MS

Top-down analysis of intact protein complexes was performed by directly sampling tissue with the nano-DESI ion source. Tissue was scanned at 1 – 2 μm/s. For subunit ion analysis, protein assemblies were dissociated to subunits either in the mass spectrometer source region (pseudo-MS^2), with collision voltage up to 250 V, or in the ion routing multipole (MS^2), after selection in the ion trap, by higher-energy collisional dissociation (HCD, normalized collision energy (NCE) in the range 20-60%). To obtain sequence information from assembly subunits multistage collisional activation was used. Subunit ions were produced by pseudo-MS^2 then selected in the ion trap and fragmented in the IRM by HCD with NCE up to 80%, which we refer to as pseudo-MS^3. Product ions were detected in the orbitrap analyser at a resolution setting of up to 240,000 (m/z 200) to isotopically resolve product ions and determine charge state.

Spectral deconvolution and protein identification

Full scan and PTCR MS^2 mass spectra were deconvoluted with the UniDec plugin in ORIGAMI to obtain intact masses.[2-3] Deconvolution parameters for Aqp0 were as follows: Bin size; m/z 0.5, charge; 5-20, m/z range; 6500 – 8000 for tetramer, 2700 – 4200 for subunits; sampling frequency; 1 Da. Other settings were left as default. For PTCR data for crystallin tetramers, deconvolution was performed as above across the full m/z range of each spectrum.

Identification of proteins from sequence ions was performed with ProSight PC (v4.1, Thermo) by importing unprocessed MS/MS data under default import settings and searching against the reference proteome for *Ovis aries* (UniProt proteome ID: UP000002356, downloaded May 2021) with a mass tolerance of 1000 Da for precursor mass and 20 ppm for product ions with "Δm mode" on. P-score[4] was used in combination with the following characteristics to inform protein identification: intact mass measurement, stoichiometry of the assembly and detection of product ions produced predominantly from cleavage at the C-terminus of aspartic acid residues and the N-terminus of proline residues, which have a high propensity for cleavage in native top-down mass spectrometry data.[5] Ions reported by Prosight were confirmed by manual investigation of the raw data using TDValidator[6] (v 1.1) and the MS-Product tool in Protein Prospector (v 6.3.1, https://prospector.ucsf.edu/prospector/cgi-bin/msform.cgi?form=msproduct). Where used, TDValidator settings were: Max PPM tolerance; 20, Sub ppm tolerance; 3, minimum score; 0.5, S/N cutoff; 3. Unspecified settings were unchanged from defaults.

SUPPORTING FIGURES

Analysis of Aquaporin-0

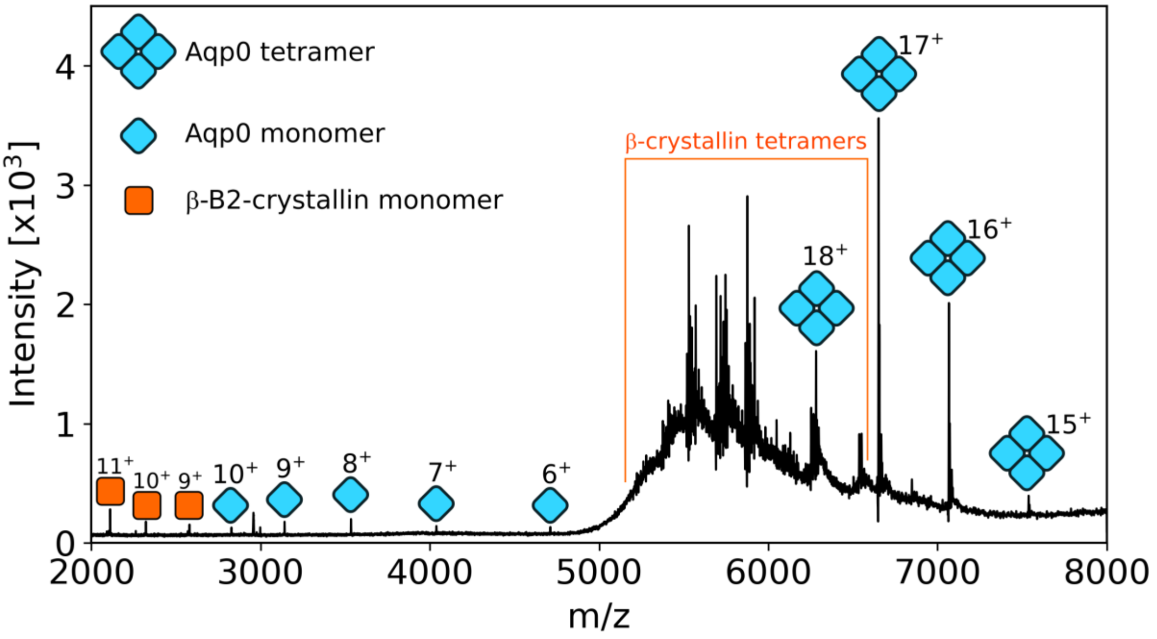

Figure S1: full scan nano-DESI mass spectrum from eye lens acquired over a wide m/z range (m/z 2000-8000) with SDV = 180V and SCV = 17%. Intact Aqp0 tetramer ions are the dominant signals. Minimal dissociation of Aqp0 and β-crystallin assemblies was observed under the instrument conditions described. Spectrum is an average of 675 scans at a resolution setting of 7500 (m/z 200).

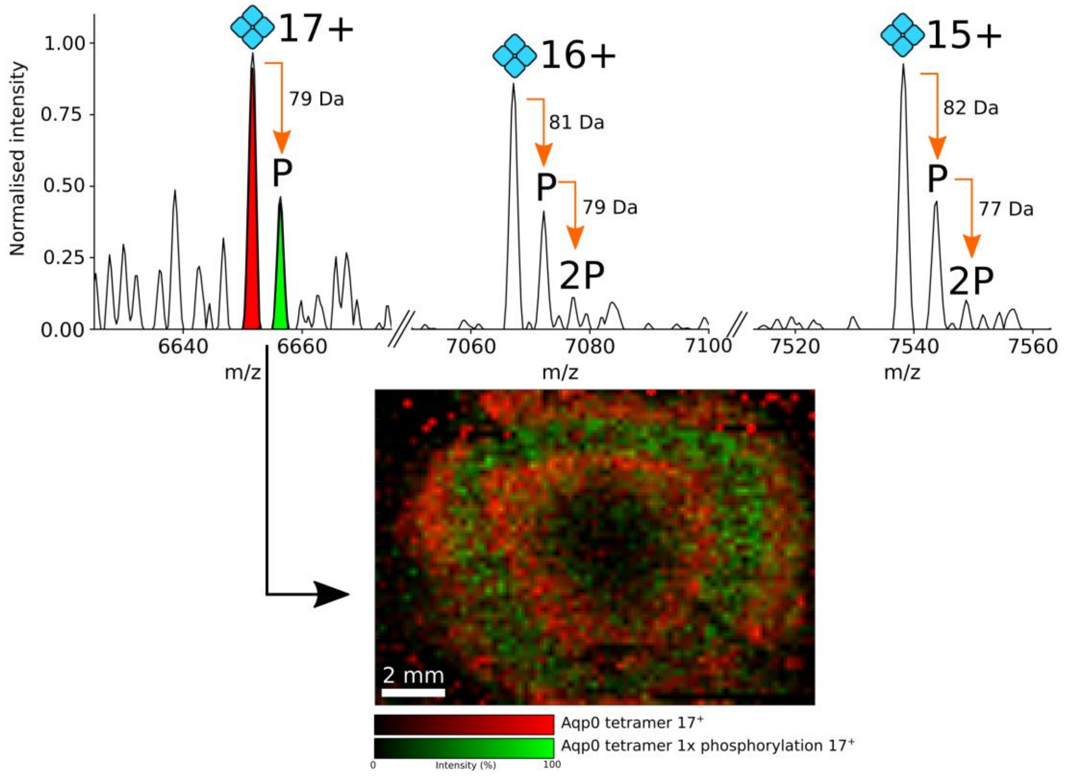

Figure S2: Full scan nano-DESI mass spectrum acquired at an orbitrap resolution of 30,000 (m/z 200). Peaks adjacent to the intact tetramer ions were observed to occur with a mass increase of approx. 80 Da, suggesting individual subunits within the tetramer were phosphorylated. The ion image (bottom) shows the intensity distribution of Aqp0 tetramer (red) versus the monophosphorylated Aqp0 tetramer (green) for the 17+ charge state.

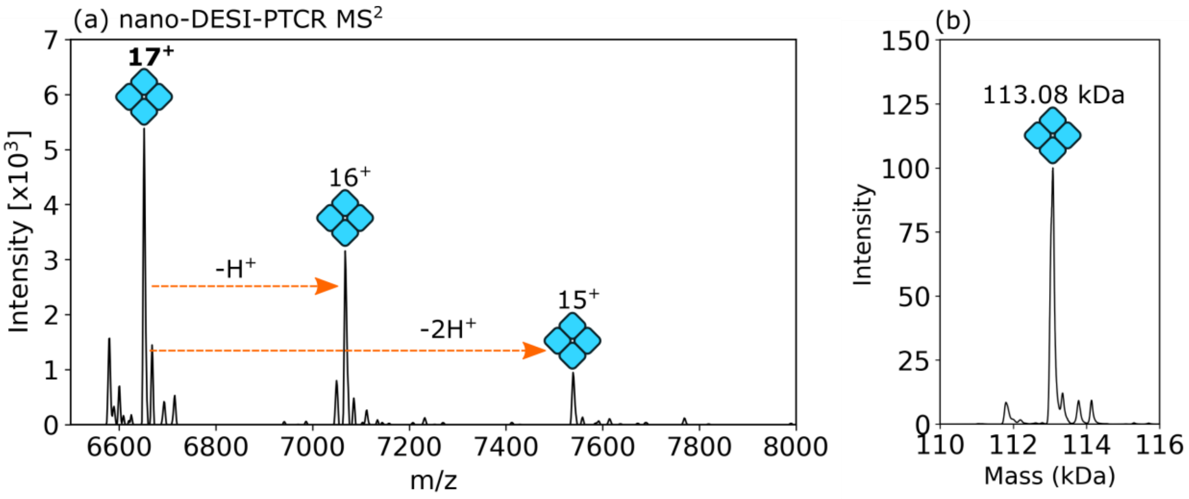

Figure S3: (a) nano-DESI-PTCR MS^2 spectrum for m/z 6652 +/- m/z 10 at an orbitrap resolution of 7500 (m/z 200). The charge reduced peaks indicated 17+ precursor ions. (b) Deconvolution of the PTCR mass spectrum provided an intact mass measurement of 113.08 kDa.

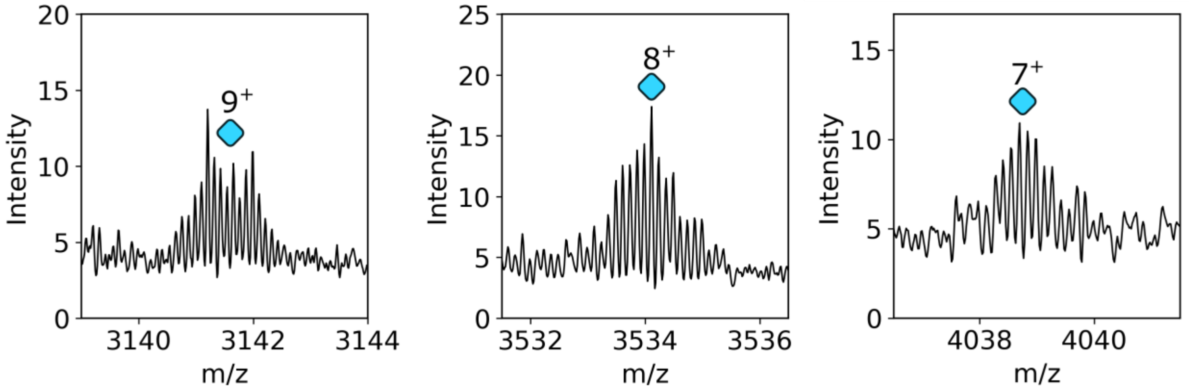

Figure S4: High-resolution (nominally 240,000 at m/z 200) mass spectra of Aqp0 subunits (charge states 9+, 8+ and 7+).

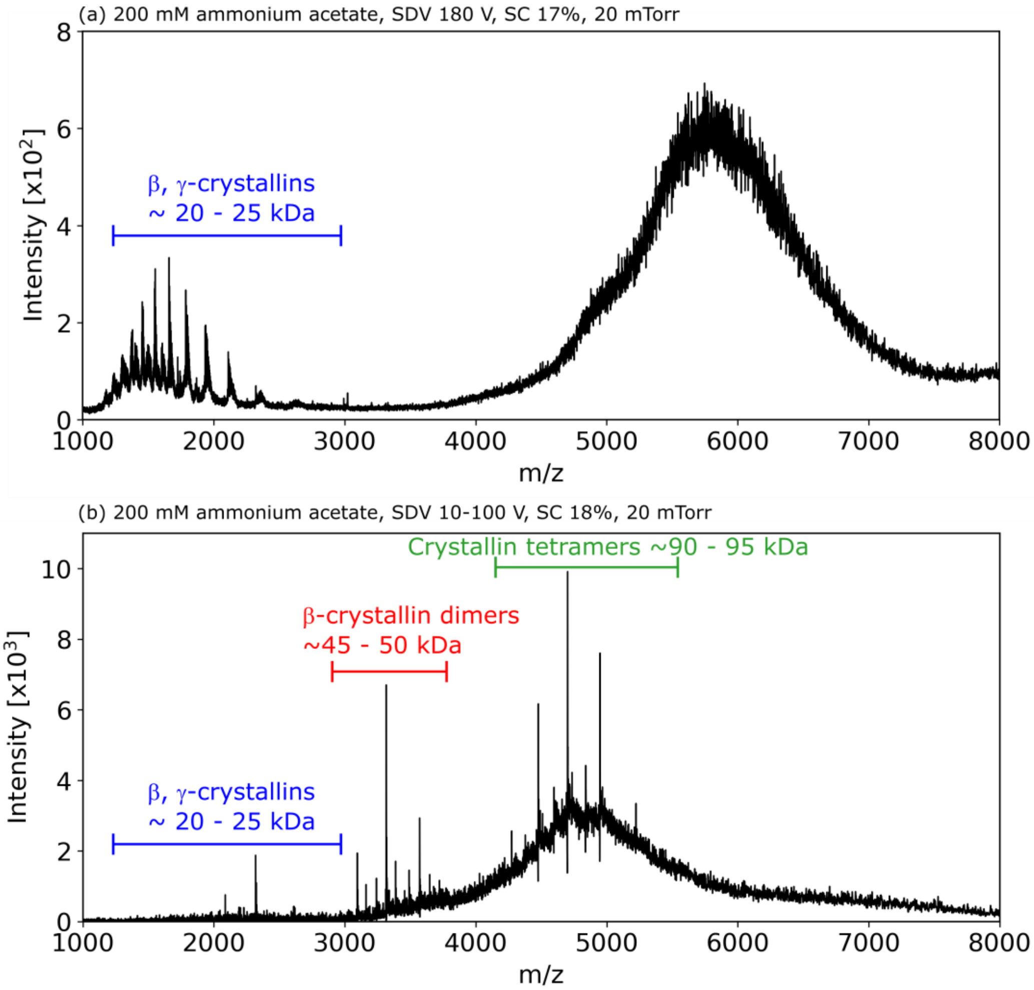

Figure S5: nano-DESI full scan mass spectra acquired from eye lens tissue using 200 mM ammonium acetate in water as sampling solvent. Detergent was not added. (a) mass spectrum acquired using the mass spectrometer conditions used for successful detection of intact Aqp0 tetramers when detergent was included; SDV = 180 V, SC 17%, IRM pressure = 20 mTorr. No intact Aqp0 tetramers or subunits were detected. Crystallin signals are likely dissociated from higher order oligomers. (b) composite (average of 502 scans) mass spectrum of data acquired with SDV 0 – 100 V, lower energy conditions than used for analysis of Aqp0 with detergent. C8E4 stabilises protein complexes, enabling higher collision energies to be used, i.e., it was possible that Aqp0 ions might be detected under less energetic conditions in the absence of the detergent, however none were observed. Crystallin monomer, dimer and tetramer signals were detected. Aqp0 tetramer or dissociated subunit signals were not detected. Higher source dissociation voltages resulted in spectra similar to (a).

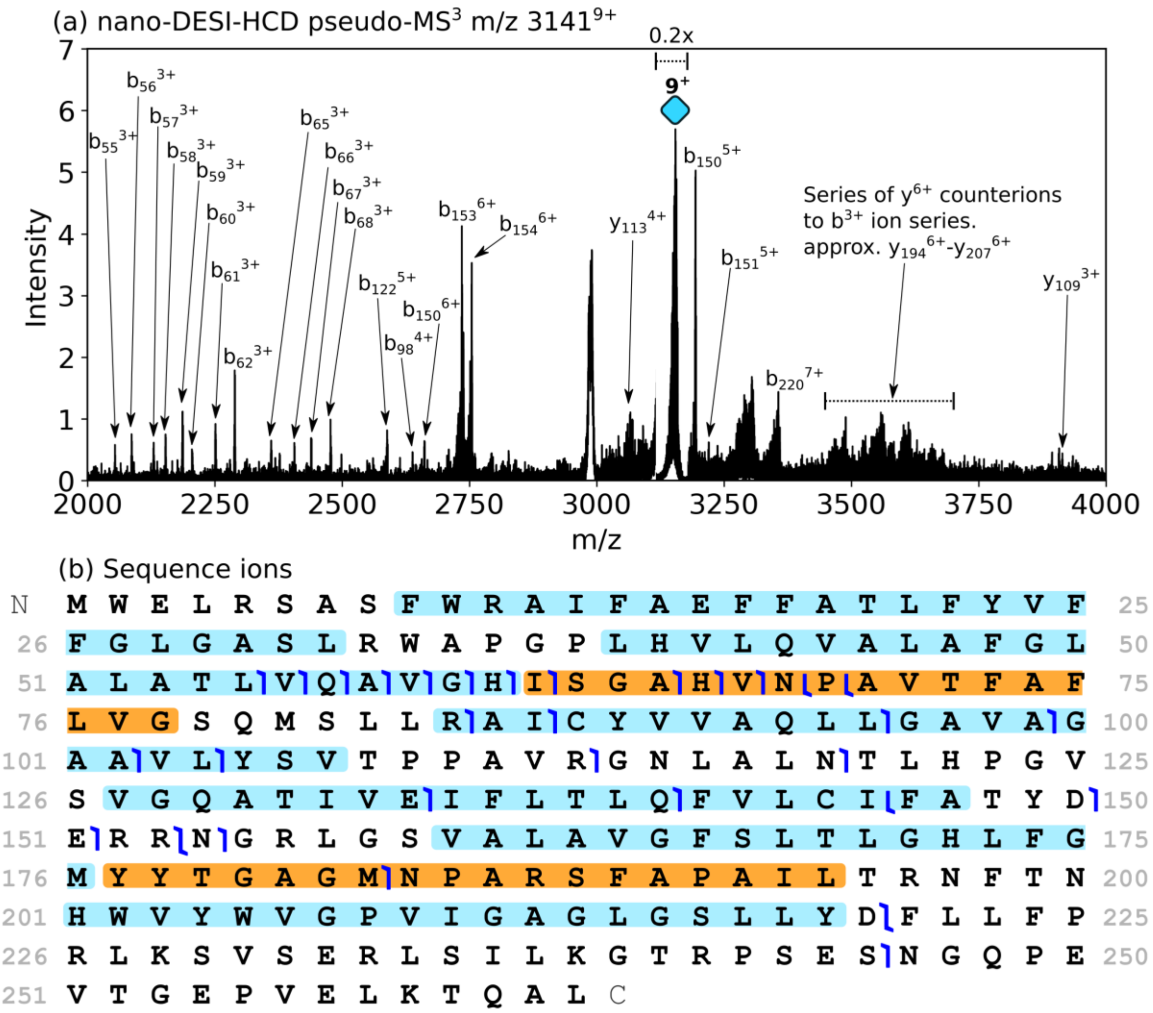

Figure S6: (a) pseudo-MS^3 mass spectrum of m/z 3141^{9+} (Aqp0 subunit ions), obtained with a NCE = 40 – 52% varied over approx. 43 minutes. Mass spectrum composed of the average signals from 1156 scans at 240,000 resolution (m/z 200). Example product ions are labelled. (b) Amino acid sequence for Aqp0 with labelling for fragment ions with a mass tolerance of 20 ppm. Residue cleavages = 11%, P-Score = 1.3e-13. Blue highlight indicates transmembrane domains, whilst orange highlight indicates intramembrane domains. In previous work, it was noted that under native-like conditions soluble protein ions would preferentially cleave at the C-terminus of Asp residues.[5] Acidic residues like Asp are rare in the sequence of multipass transmembrane proteins, and Aqp0 is deficient in Asp-residues, featuring only two throughout its 263-residue sequence.[7] Signals indicative of cleavage at these residues are evident in the product ion spectrum (b_{150}, b_{220}), with the most abundant product ion being b_{150}^{5+}. An interesting sequence of backbone fragments spans residues 55 – 70. This region of the sequence passes from a transmembrane helix to an intramembrane helix.

Table S1: Aqp0 sequence ions within 20 ppm of the theoretical mass.

Name	Theoretical Mass	Observed Mass	Mass Difference (Da)	Mass Difference (ppm)
**b55**	6157.2788	6157.2274	-0.0514	-8.3
**b56**	6256.3472	6256.2969	-0.0504	-8.0
**b57**	6384.4058	6384.3702	-0.0356	-5.6
**b58**	6455.4429	6455.3752	-0.0677	-10.5
**b59**	6554.5113	6554.4785	-0.0328	-5.0
**b60**	6611.5328	6611.4748	-0.0580	-8.8
**b61**	6748.5917	6748.5368	-0.0549	-8.1
**b62**	6861.6758	6861.6060	-0.0697	-10.2
**b65**	7076.7664	7076.7088	-0.0575	-8.1
**b66**	7213.8253	7213.7696	-0.0557	-7.7
**b67**	7312.8937	7312.8375	-0.0562	-7.7
**b68**	7426.9377	7426.8999	0.0378	-5.1
**b85**	9244.9228	9244.8391	-0.0837	-9.1
**b87**	9429.0440	9428.8686	-0.1754	-18.6
**b95**	10318.5171	10318.4844	-0.0327	-3.2
**b98**	10545.6456	10545.5880	0.0576	-5.5
**b99**	10616.6812	10616.6227	-0.0585	-5.5
**b102**	10815.7769	10815.6992	-0.0777	-7.2
**b104**	11027.9294	11027.9017	-0.0277	-2.5
**b113**	11998.4530	11998.3630	-0.0900	-7.5
**b119**	12580.7655	12580.7347	-0.0308	-2.5
**b122**	12931.9584	12931.9416	0.0168	-1.3
**b134**	14069.5591	14069.3986	-0.1606	-11.4
**b140**	14784.9860	14784.8940	-0.0919	-6.2
**b150**	15957.5436	15957.3956	-0.1480	-9.3
**b151**	16086.5862	16086.5114	-0.0748	-4.6
**b153**	16398.7884	16398.6615	-0.1269	-7.7
**b154**	16512.8334	16512.8130	0.0204	-1.2
**b183**	19440.3139	19440.1452	-0.1686	-8.7
**b220**	23510.7599	23510.3302	-0.4297	-18.3
**b245**	26336.0372	26335.8659	-0.1713	-6.5
**y43**	4750.6028	4750.5696	0.0332	-7.0
**y113**	12287.4744	12287.4056	0.0688	-5.6
**y118**	12884.7164	12884.8610	0.1446	11.2
**y194**	20721.0271	20720.8725	-0.1546	-7.5
**y195**	20818.0799	20817.8857	-0.1941	-9.3

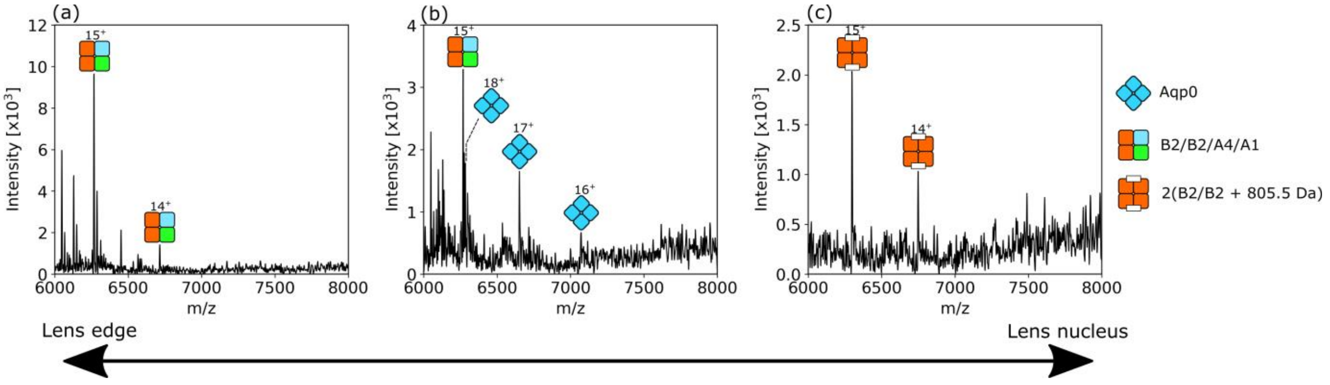

Figure S7: Example average mass spectra (orbitrap resolution 7500 at m/z 200) from a nano-DESI line scan in eye lens showing signals for intact membrane and soluble protein assemblies. (a) Lens edge (15 scans) (b) midway between lens edge and lens nucleus (18 scans) and (c) lens nucleus (20 scans).

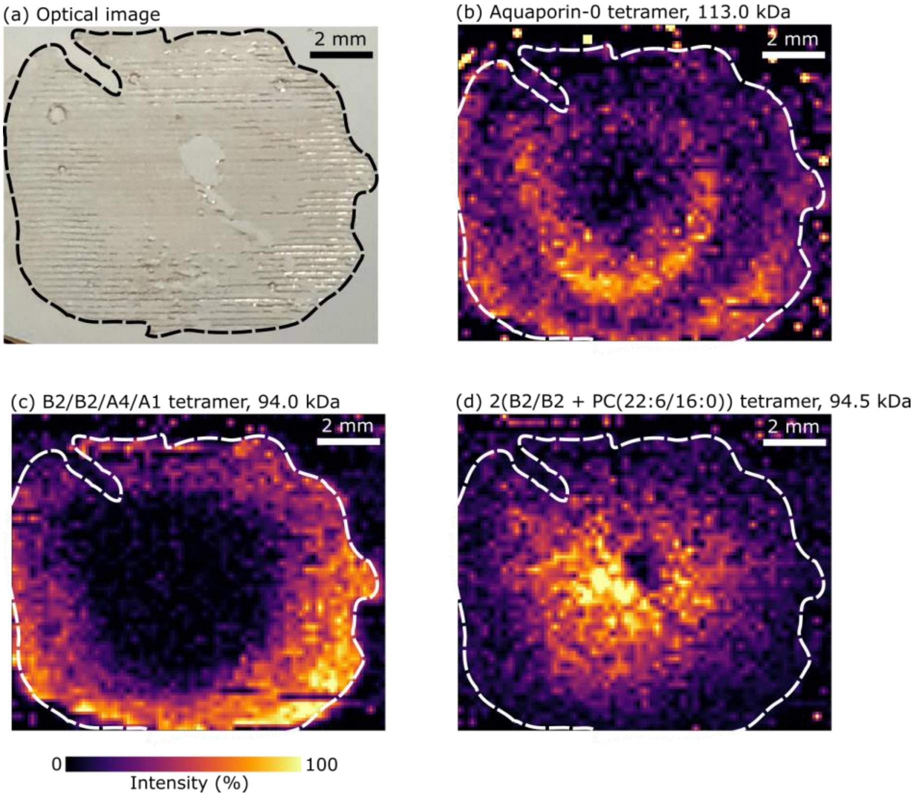

Figure S8: (a) Optical image of the analysed lens tissue section. Lines are visible from the nano-DESI probe. (b) Ion image of Aqp0 tetramer (113.1 kDa, m/z 6651.5^{17+} ± 0.2. (c) Ion image of B2/B2/A4/A1 crystallin tetramer (94.0 kDa, m/z 6267.3^{15+} ± 0.2). (d) Ion image of 2(B2/B2 + PC (22:6/16:0)) crystallin tetramer (94.5 kDa, m/z 6299.2^{15+} ± 0.2). Ion images are TIC normalised with a linear intensity scale. Duplicate analysis of the lens in Figure 2.

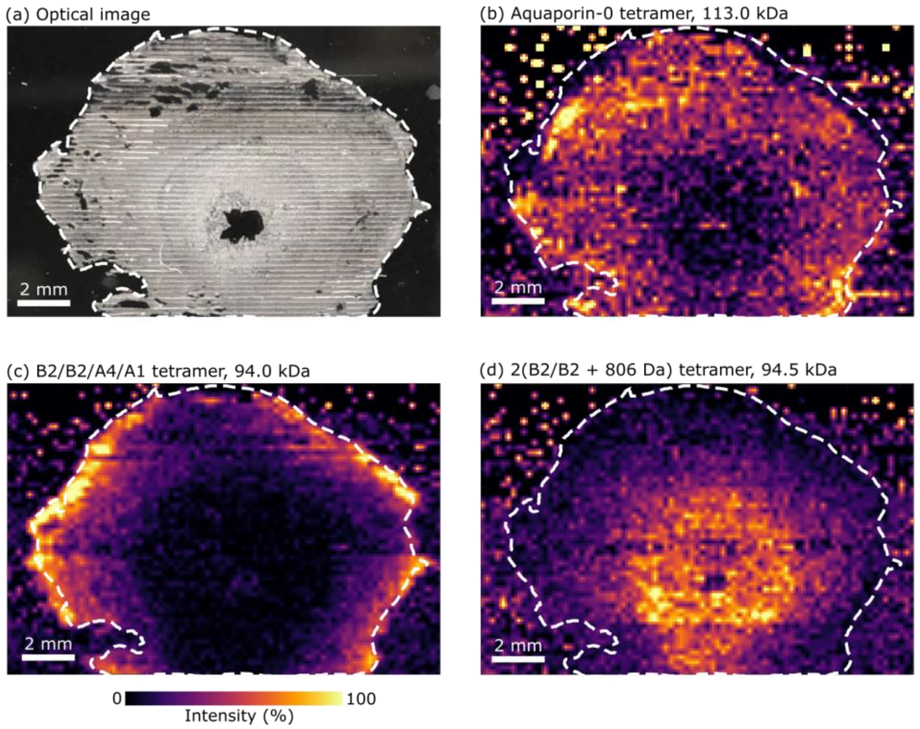

Figure S9: (a) Optical image of the analysed lens tissue section. Lines are visible from the nano-DESI probe. (b) Ion image of Aqp0 tetramer (113.1 kDa, m/z 6651.5^{17+} ± 0.2. (c) Ion image of B2/B2/A4/A1 crystallin tetramer (94.0 kDa, m/z 6267.3^{15+} ± 0.2). (d) Ion image of 2(B2/B2 + PC (22:6/16:0)) crystallin tetramer (94.5 kDa, m/z 6299.2^{15+} ± 0.2). Ion images are TIC normalised with a linear intensity scale. Duplicate analysis of the lens in Figure S9.

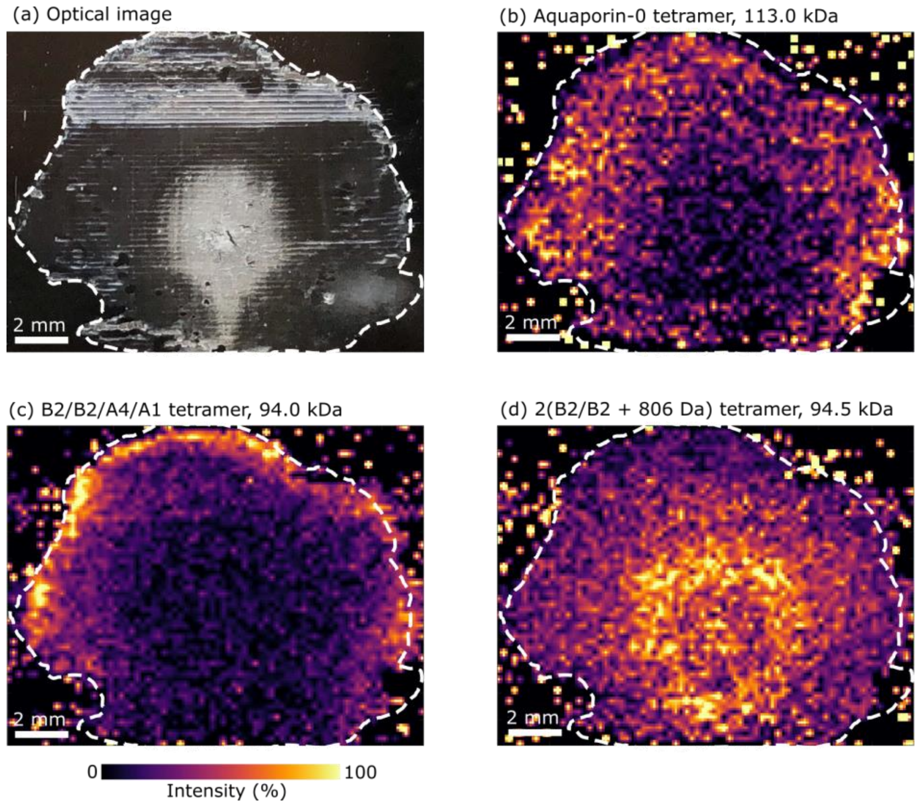

Figure S10: (a) Optical image of the analysed lens tissue section. Lines are visible from the nano-DESI probe. (b) Ion image of Aqp0 tetramer (113.1 kDa, m/z 6651.5^{17+} ± 0.2. (c) Ion image of B2/B2/A4/A1 crystallin tetramer (94.0 kDa, m/z 6267.3^{15+} ± 0.2). (d) Ion image of 2(B2/B2 + PC (22:6/16:0)) crystallin tetramer (94.5 kDa, m/z 6299.2^{15+} ± 0.2). Ion images are TIC normalised with a linear intensity scale. Duplicate analysis of the lens in Figure S8.

β-Crystallins

Table S2: β-crystallin monomers

Name	Uniprot accession	PTMs	Calculated Average mass (Da)	Measured average mass (Da)[a]	Mass difference (Da)
**β-B2**	W5QCG5	-M, N-term. Acet.	23208.8576	23208.2973 ± 0.06	0.5603
**β-A4**	A0A6P7EYH2	-M, N-term. Acet.	22411.9004	22410.7813 ± 0.01	1.119
**β-A1**	W5P9A5	N-term. Acet	25173.8812	25173.1590 ± 0.03	0.7222

[a] one standard deviation from three measurements indicated.

Tetramers

B2/B2/A4/A4

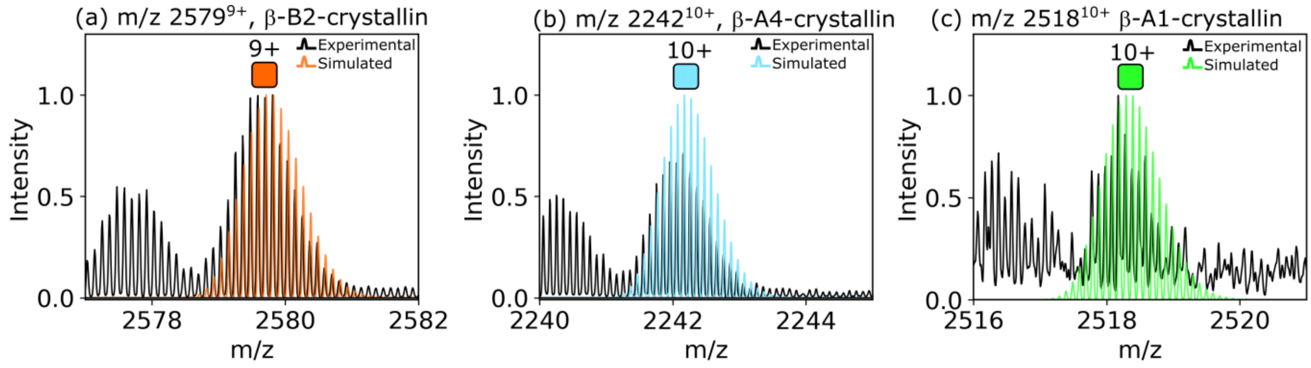

Figure S11: Simulated mass spectra generated at a resolution of 76000 (approximately equivalent to the resolution of the measured signals from an orbitrap resolution setting of 240,000 at m/z 200) were overlayed on the HCD product ion signals from the tetramer with m/z 5876^{16+}; (a) N-terminally acetylated β-B2-crystallin,(b) N-terminally acetylated β-A4-crystallin and (c) N-terminally acetylated β-A1-crystallin. Simulated spectra were generated from sequences of proteins in Table S2.

Dimers

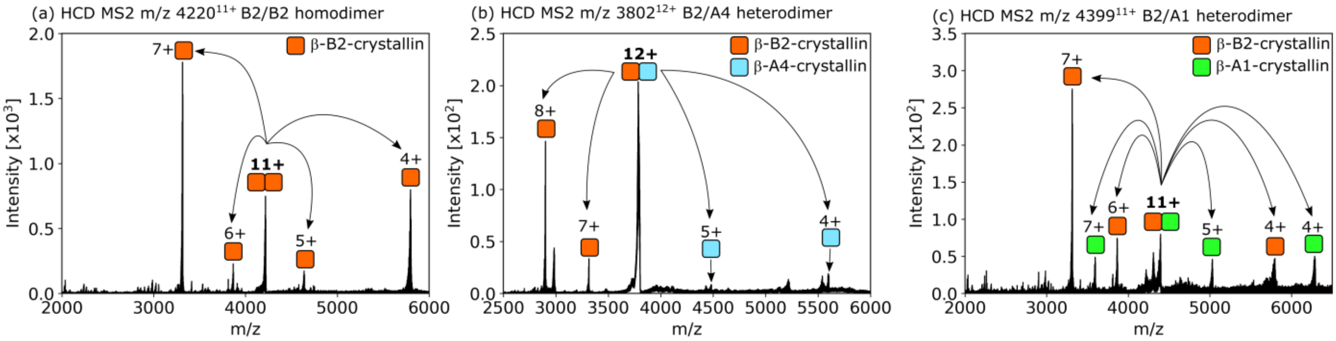

Figure S12: the subunit composition of β-crystallin dimers with relevance to discussed β-crystallin tetramers. Nano-DESI-HCD MS^2 spectra for (a) B2/B2 homodimer (NCE=40%), (b) B2/A4 heterodimer (NCE=35%) and (c) B2/A1 heterodimer (NCE=42%). SDV was set to 150 V to dissociate higher-order oligomers. SCV was set to 8% to transmit proteins approx. 45 kDa.

Crystallin monomer identification

Beta-B2-crystallin

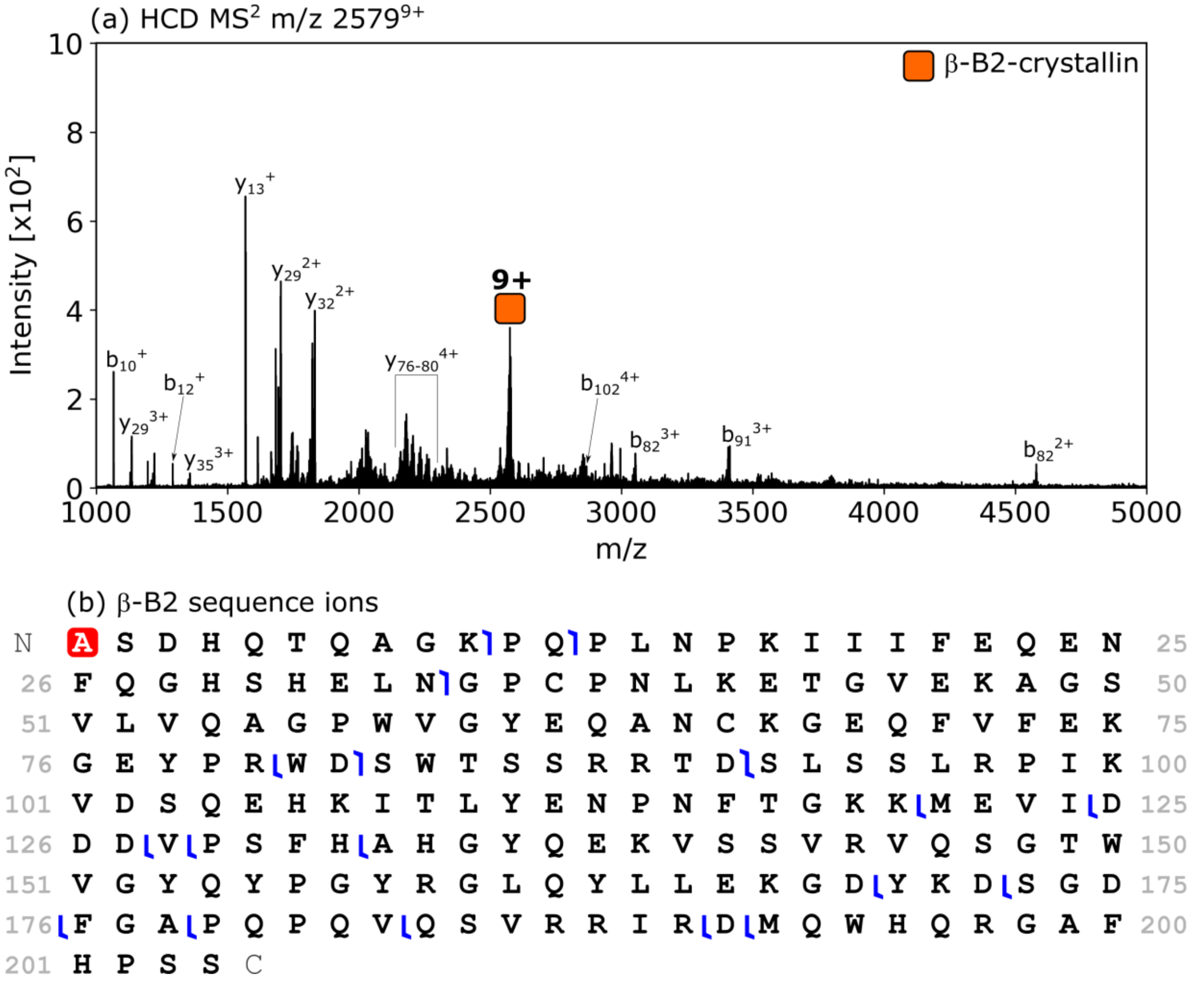

Figure S13: (a) nano-DESI HCD MS2 spectrum for β-B2-crystallin acquired with a NCE = 38-41%. (b) Sequence of β-B2-crystallin. Matched sequence ions are labelled within a mass tolerance of 20 ppm. The N-terminus is acetylated, highlighted in red. Residue cleavages = 9%, P-score = 4.9e-6. 13 of 18 fragment ions are explained by cleavage at the C-terminus of D or N-terminus of P.

Table S3: sequence ions for beta-B2-crystallin matched within a mass tolerance of 20 ppm.

Name	Theoretical Mass (Da)	Observed Mass (Da)	Mass Difference (Da)	Mass Difference (ppm)
**b10**	1065.4839	1065.4791	-0.0048	-4.5
**b12**	1290.5953	1290.5916	-0.0036	-2.8
**b34**	3875.8979	3875.8511	-0.0468	-12.1
**b82**	9154.4259	9154.3309	-0.0950	-10.4
**y13**	1567.7103	1567.7007	-0.0096	-6.1
**y14**	1682.7372	1682.7285	-0.0086	-5.1
**y21**	2578.2836	2578.2454	-0.0382	-14.8
**y26**	3127.5747	3127.5426	-0.0321	-10.3
**y29**	3402.7017	3402.6727	-0.0290	-8.5
**y32**	3661.7821	3661.7590	-0.0231	-6.3
**y35**	4067.9673	4067.9462	-0.0211	-5.2
**y72**	8268.0490	8267.9256	-0.1234	-14.9
**y76**	8736.2611	8736.2021	-0.0591	-6.8
**y77**	8835.3295	8835.2101	-0.1195	-13.5
**y80**	9180.4103	9180.3298	-0.0806	-8.8
**y84**	9652.6459	9652.5989	-0.0470	-4.9
**y113**	12963.4132	12963.3478	-0.0654	-5.0
**y124**	14341.0194	14340.8561	-0.1633	-11.4

Beta-A4-crystallin

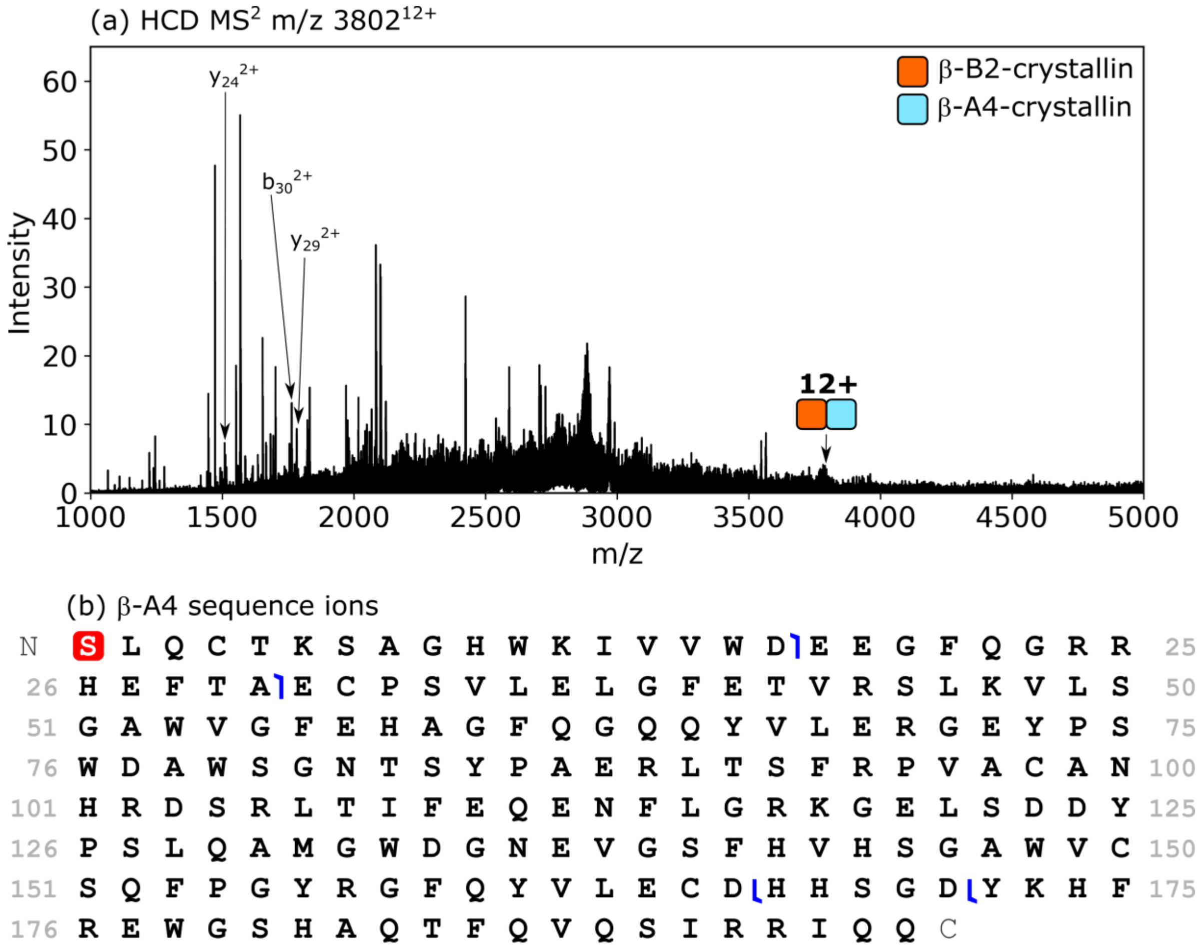

Figure S14: (a) nano-DESI HCD MS^2 spectrum of the B2/A4 dimer. NCE = 60% was used to fragment the peptide backbone of both monomers. Sequence ions for β-A4-crystallin are labelled. (b) Sequence ions for β-A1-crystallin matched within a mass tolerance of 20 ppm. The N-terminus is acetylated, indicated by the red highlight. The P-score = 0.57 and is influenced by the presence of fragment ions from β-B2-crystallin in the same spectrum (P-score = 0.022) because of fragmenting the dimer. 3 of 4 β-A4 fragment ions are explained by cleavage at the C-terminus of D-residues.

Table S4: Sequence ions for beta-A4-crystallin within a mass tolerance of 20 ppm.

Name	Theoretical Mass (Da)	Observed Mass (Da)	Mass Difference (Da)	Mass Difference (ppm)
**b17**	1980.9883	1980.9696	0.0187	-9.4
**b30**	3525.7000	3525.6764	-0.0236	-6.7
**y24**	3029.5485	3029.5213	-0.0272	-9.0
**y29**	3562.7467	3562.7123	-0.0344	-9.7

Beta-A1-crystallin

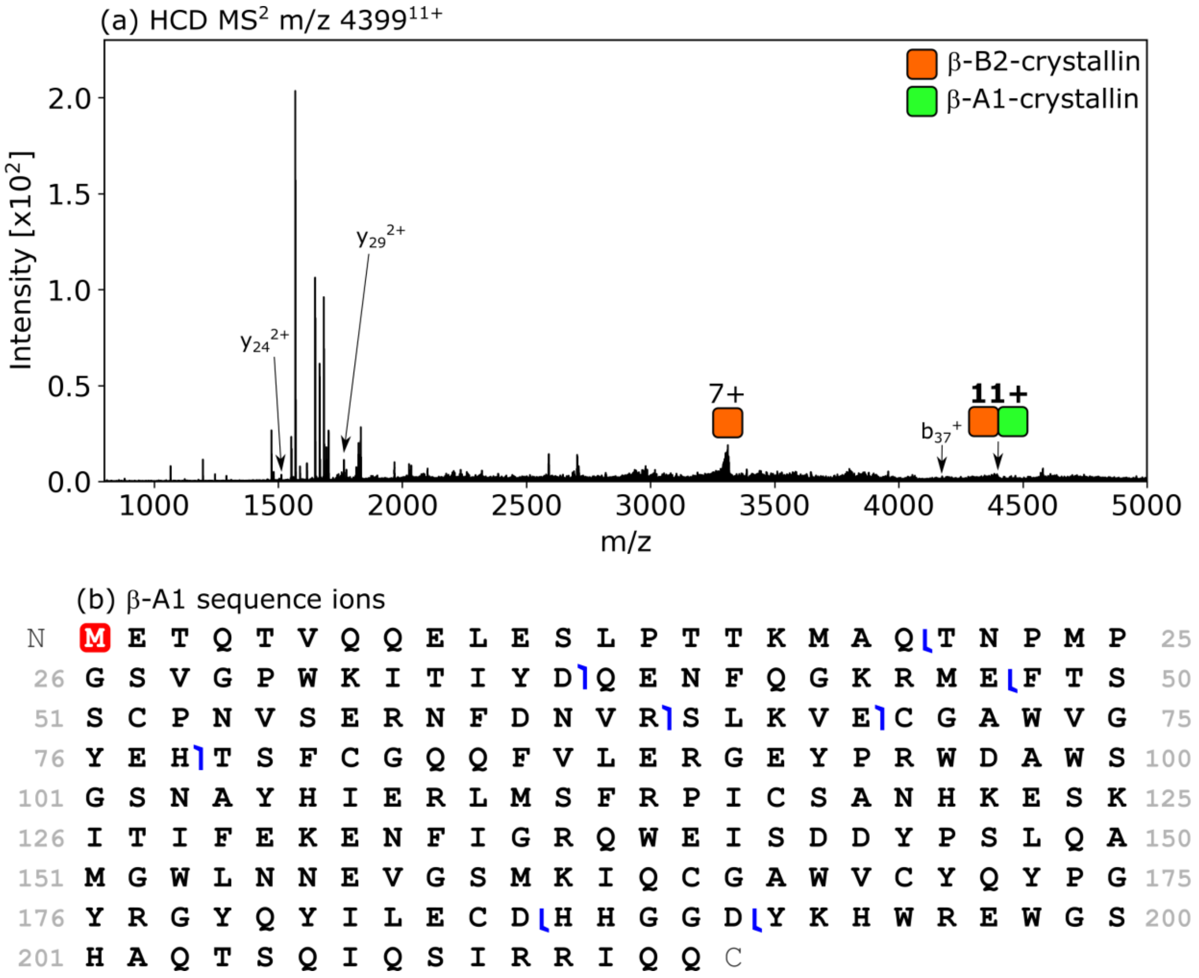

Figure S15: (a) nano-DESI HCD MS2 spectrum of the B2/A1 dimer. NCE = 42% was used to fragment the peptide backbone of both monomers. Sequence ions for β-A1-crystallin are labelled. (b) Sequence ions for β-A1-crystallin matched within a mass tolerance of 20 ppm. The N-terminus is acetylated. The P-score = 0.16 and is influenced by the presence of multiple fragment ions from β-B2-crystallin in the same spectrum (P-score = 1e-8) as a result of fragmenting the dimer. 3 of 8 ions were detected as the result of cleavage at the C-terminus of D-residues. Note the similarity of the C-terminal sequences of β-A4 (Figure S13) and β-A1 and that fragment ions y24 and y29 are observed in each, corresponding to cleavage at D-residues (equivalent ions in each crystallin differ in mass).

Table S5: Sequence ions for beta-A1-crystallin within a mass tolerance of 20 ppm.

Name	Theoretical Mass (Da)	Observed Mass (Da)	Mass Difference (Da)	Mass Difference (ppm)
**b37**	4173.0111	4173.0027	0.0084	-2.0
**b64**	7373.4624	7373.5739	0.1115	15.1
**b69**	7929.7845	7929.7986	0.0141	1.8
**b78**	8932.1862	8932.2306	0.0443	5.0
**y24**	3022.5386	3022.5181	-0.0205	-6.8
**y29**	3525.7263	3525.6803	-0.0460	-13.1
**y168**	19736.2342	19736.3767	0.1426	7.2
**y195**	22840.7189	22840.9121	0.1931	8.5

References

[1] G. Robichaud, K. P. Garrard, J. A. Barry, D. C. Muddiman, *J Am Soc Mass Spectrom,* **2013**, *24*, 718-721.

[2] M. T. Marty, A. J. Baldwin, E. G. Marklund, G. K. Hochberg, J. L. Benesch, C. V. Robinson, *Anal Chem,* **2015**, *87*, 4370-4376.

[3] L. G. Migas, A. P. France, B. Bellina, P. E. Barran, *Int J Mass Spectrom,* **2018**, *427*, 20-28.

[4] F. Meng, B. J. Cargile, L. M. Miller, A. J. Forbes, J. R. Johnson, N. L. Kelleher, *Nature Biotechnology,* **2001**, *19*, 952-957.

[5] A. Ives, T. Su, K. R. Durbin, B. P. Early, H. dos Santos Seckler, R. T. Fellers, R. D. LeDuc, L. F. Schachner, S. M. Patrie, N. L. Kelleher, *J Am Soc Mass Spectrom,* **2020**.

[6] L. Fornelli, K. Srzentić, R. Huguet, C. Mullen, S. Sharma, V. Zabrouskov, R. T. Fellers, K. R. Durbin, P. D. Compton, N. L. Kelleher, *Anal Chem,* **2018**, *90*, 8421-8429.

[7] J. A. Baker, W. C. Wong, B. Eisenhaber, J. Warwicker, F. Eisenhaber, *BMC Biol,* **2017**, *15*, 66.
